# Notes on the *Stenus cirrus* group of Zhejiang, East China, with descriptions of two new species (Coleoptera, Staphylinidae)

**DOI:** 10.3897/zookeys.684.13524

**Published:** 2017-07-11

**Authors:** Sheng-Nan Liu, Liang Tang, Rong-Ting Luo

**Affiliations:** 1 Department of Biology, Shanghai Normal University, 100 Guilin Road, 1st Educational Building 323 Room, Shanghai, 200234 P. R. China

**Keywords:** China, Coleoptera, new species, Staphylinidae, *Stenus cirrus* group, Zhejiang

## Abstract

Two new *Stenus* species of the *cirrus* group collected from Zhejiang Province, East China, are described, *S. wuyanlingus* Liu, Tang & Luo, **sp. n.**, *S. yuyimingi* Liu, Tang & Luo, **sp. n.** and a new distributional locality for *S. ovalis* Tang, Li & Zhao, 2005 was discovered. The diagnostic characters of the new species are illustrated, and a key to species of the group from Zhejiang Province is provided.

## Introduction

The *Stenus cirrus* group is a large group with 73 species worldwide. Among them, 56 species are known from China (Tang et al. 2008; Puthz 2009; Yu et al. 2014; Liu and Tang 2017) and seven species are known from Zhejiang: *S. cirrus* Benick, L., 1940, *S. guangxiensis* Rougemont, 1984, *S. ovalis* Tang, Li & Zhao, 2005, *S. zhulilongi* Tang & Puthz, 2008, *S. lijinweni* Tang & Puthz, 2008, *S. jinlongshanus* Tang & Puthz, 2008 and *S. shenshanjiai* Tang & Puthz, 2008. The members of the group are characterized by the oval paraglossae and the presence of long and suberect setae on abdomen. Recently, two new species of the *Stenus cirrus* group from Zhejiang Province were found in our collections and they will be described in this paper.

## Material and methods

The specimens examined in this paper were mainly collected at various locations in Zhejiang, East China by sifting leaf litter in broad leaf forests. Specimens were killed with ethyl acetate and dried. For examination of the male and female genitalia, the apical three abdominal segments were detached from the body after softening in hot water. The aedeagi, together with other dissected parts, were mounted in Euparal (Chroma Gesellschaft Schmidt, Koengen, Germany) on plastic slides. Photos of sexual characters were taken with a Canon G9 camera attached to an Olympus CX31 microscope; habitus photos were taken with a Canon macro photo lens MP-E 65 mm attached to a Canon EOS7D camera and stacked with Zerene Stacker.

The type specimens treated in this study are deposited in the following public and private collections:

**SHNU** Department of Biology, Shanghai Normal University, P. R. China

**cPut** Private collection V. Puthz, Schlitz, Germany

The measurements of proportions are abbreviated as follows:

**BL** body length, measured from the anterior margin of the clypeus to the posterior margin of abdominal tergite X

**FL** forebody length, measured from the anterior margin of the clypeus to the apicolateral angle of elytra

**HW** width of head including eyes

**PW** width of pronotum

**EW** width of elytra

**PL** length of pronotum

**EL** length of elytra, measured from humeral angle

**SL** length of elytral suture

## Taxonomy

### Stenus wuyanlingus

Taxon classificationAnimaliaColeopteraStaphylinidae

Liu, Tang & Luo sp. n.

http://zoobank.org/0F0F88C6-8EA2-4316-B391-B24D295BCF1A

Figs 1 , 2 , 7–13

#### Type material.

**CHINA: Zhejiang: Holotype**: ♂, glued on a card with labels as follows: “China: Zhejiang Prov. Taishun County, Wuyanling, 27°42’N, 119°41’E, alt. 1550 m. 6.V.2012, ZHU Jian-Qing leg”. “Holotype / *Stenus wuyanlingus* / Liu & Tang” [red handwritten label] (SHNU). **Paratypes**: 6♂♂5♀♀, same data as for the holotype. (1♂1♀ in cPut, remainder in SHNU).

**Figures 1–6.**
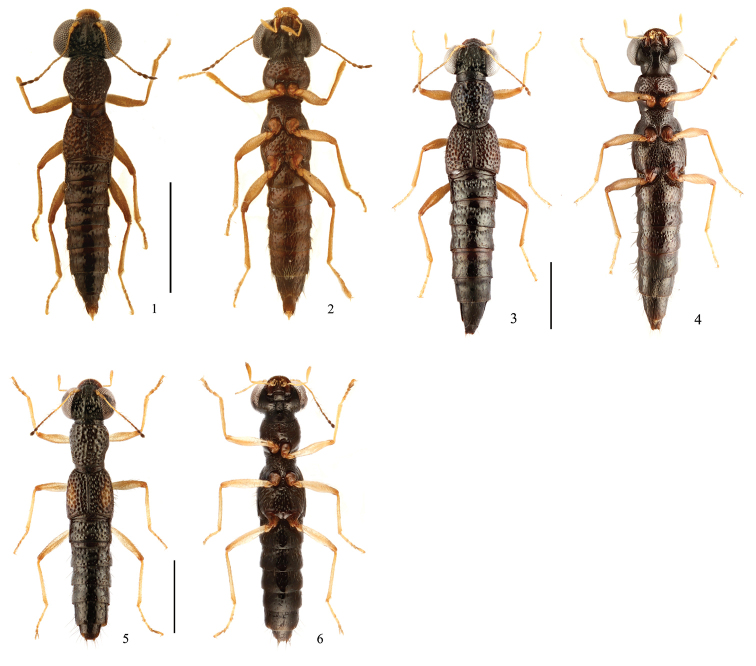
Habitus. **1, 2** *Stenus wuyanlingus* sp. n. **3, 4** *Stenus yuyimingi* sp. n. **5, 6** *Stenus ovalis*. Scale bars: 1 mm.

#### Description.

Brachypterous. Head black, pronotum and abdomen blackish brown, elytra light brown with median portion more or less lighter. Antennae, maxillary palpi and legs yellowish brown except antennal club infuscate. Labrum reddish brown.

BL: 2.7–3.1 mm, FL: 1.4–1.6 mm. HW: 0.63–0.71 mm, PL: 0.45–0.49 mm, PW: 0.43–0.51 mm, EL: 0.44–0.52 mm, EW: 0.48–0.61 mm, SL: 0.32–0.40 mm.

**Head** 1.21–1.30 times as wide as elytra; interocular area with two deep longitudinal furrows, median portion convex, slightly extending a little beneath the level of inner eye margins, with a impunctate line along midline; punctures round, larger and sparser in median portion than those near inner margins of eyes, diameter of large punctures about as wide as apical cross section of antennal segment II; interstices between punctures distinctly reticulated, varied from narrower than half the diameter of punctures to a little narrower than diameter of punctures except those along the midline of convex median portion, which may be slightly wider than diameter of punctures. Paraglossae oval.

**Pronotum** 0.94–1.04 times as long as wide; disk uneven, with distinct median longitudinal furrow almost throughout; punctures confluent, varied in size, mostly smaller than large punctures of head; interstices reticulated, narrower than diameter of punctures except those in median area, which may be much wider than diameter of punctures.

**Elytra** 0.81–0.95 times as long as wide; disk with shallow sutural impression and humeral impression; punctures confluent, slightly larger than those on pronotum in average; interstices rarely reticulated, narrower than half the diameter of punctures.

**Legs** with tarsomeres IV strongly bilobed.

**Abdomen** cylindrical; paratergites very narrow with few punctures, present only in segment III, tergites and sternites totally fused in segment IV–VI, posterior margin of tergite VII with indistinct palisade fringe; punctation round to elliptic, gradually becoming smaller and sparser posteriad; interstices smooth, mostly wider than diameter of punctures except those on basal impressions of tergites III–V, which may be distinctly narrower than half the diameter of punctures.

**Figures 7–13.**
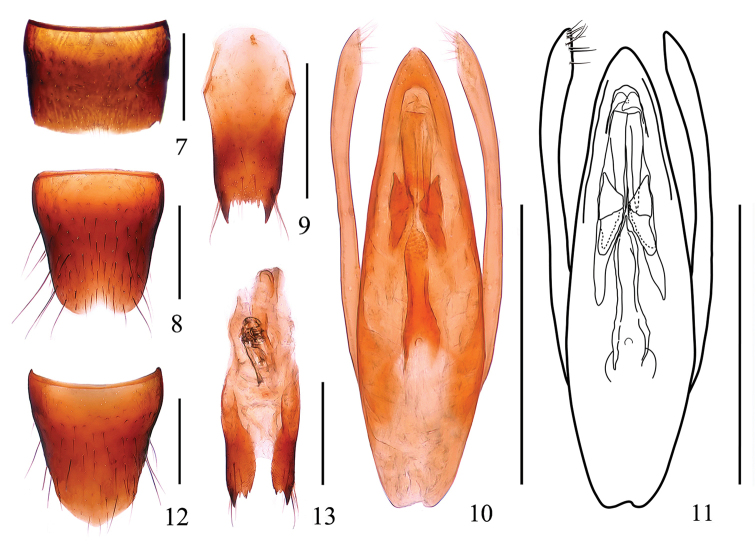
*Stenus wuyanlingus*. **7** male sternite VII **8** male sternite VIII **9** male sternite IX **10, 11** aedeagus **12** female sternite VIII **13** valvifers and spermatheca. Scale bars: 0.25 mm.

***Male.*** Sternite VII (Fig. 7) with inconspicuous emargination at middle of posterior margin; sternite VIII (Fig. 8) with shallow emargination at middle of posterior margin; sternite IX (Fig. 9) with long apicolateral projections, posterior margin serrate. Aedeagus (Figs 10–11) widest at basal third and tapering towards the apex; median longitudinal bands each with ventral band very long, straight, narrowed apically; lateral longitudinal bands short; expulsion clasps each with anterior plate distinctly separated from the posterior plate, rounded apically, posterior plate pointed posteriorly; copulatory tube moderately long, rather stout, with basal chamber submembranous, main tube distinctly divided into basal tube and apical tube, the basal tube gently constricted near the middle, apical tube very thin; parameres longer than median lobe, each with 8–11 setae on apico-internal margins.

***Female.*** Sternite VIII (Fig. 12) with posterior margin weakly pointed at middle; spermatheca (Fig. 13) strongly sclerotized, spermathecal duct with multiple bends.

#### Distribution.

China (Zhejiang).

#### Remarks.

The new species can be easily recognized among Chinese members of the group by the smaller size and reticulations of head and pronotum. It can be distinguished from other species by its sparser punctation along in median area of head and pronotum, and the lighter color of elytra.

#### Etymology.

The specific name is derived from the type locality of this species.

### Stenus yuyimingi

Taxon classificationAnimaliaColeopteraStaphylinidae

Liu, Tang & Luo sp. n.

http://zoobank.org/40B0916F-3286-4CBA-A7C8-3909E9C019C2

Figs 3 , 4 , 14–20

#### Type material.

**CHINA: Zhejiang: Holotype**: ♂, glued on a card with labels as follows: “China: S. Zhejiang, Lishui City, Longquan Fengyangshan N.R., forest nr. Xiabian Vil., 27°55’58’’N, 119°10’57’’E, mixed leaf litter, sifted, 690–780 m, 9.X.2013, Z. Peng, Y.-M. Yu, Z.-W. Yin leg”. “Holotype / *Stenus yuyimingi* / Liu & Tang” [red handwritten label] (SHNU). Paratypes: 1♂, same data as for the holotype, but Mihougu, 27°55’0.18’’N, 119°11’52.91’’E, near stream, 1116 m, 03.V.2016, Jiang, Liu & Zhou leg (SHNU); 1♂, same data as for the holotype, but Datianping, 27°54’33 ‘’-55’18’’N, 119°10’20’’-17’’E, mixed litter, moss, sifted, 1170–1300 m. 7.X.2013, Feng, Peng, Yu, Yin leg. (cPut); 1♂, same data as for the holotype, but Jitou Vil., 27°55’58’’N, 119°12’44’’E, fern, silvergrass, fir, sifted, ca, 1050 m, 9.X.2013, Z Peng, Y.-M. Yu, Z.-W. Yin leg. (SHNU); 1♂1♀, same data as for the holotype, but Da-Tian-Ping 27°54’36‘’N, 119°10’20’’E, bush leaf, moss, ferns, sifted & beating, 1320 m. 27.IV.2014, Peng, Song, Yan, Yin & Yu leg. (SHNU); 1♀, same data as for the holotype (SHNU); 1♀, same data as for the holotype, but Luao Vil., 27°55’00’’N, 119°11’53’’E, moss, fern, bamboo, bush, sifted, 1130 m, 4.X.2013, Z Peng, Y.-M. Yu, Z.-W. Yin leg. (SHNU).

#### Description.

Brachypterous. Head black, pronotum and elytra brown, each elytron with median portion inconspicuously lighter, abdomen dark brown. Antennae, maxillary palpi and legs yellowish brown except antennal club infuscate. Labrum reddish brown.

BL: 3.6–4.4 mm, FL: 1.8–2.1 mm. HW: 0.77–0.86 mm, PL: 0.61–0.69 mm, PW: 0.56–0.66 mm, EL: 0.70–0.78 mm, EW: 0.70–0.85 mm, SL: 0.52–0.59 mm.

**Head** 1.02–1.09 times as wide as elytra; interocular area with two deep longitudinal furrows, median portion convex, extending to the level of inner eye margins; punctures round, distinctly larger and sparser in median portion than those near inner margins of eyes, diameter of large punctures slightly wider than apical cross section of antennal segment II; interstices smooth, narrower than half the diameter of punctures except those along the midline of the median portion, which may be as wide as the diameter of punctures. Paraglossae oval.

**Pronotum** 0.98–1.10 times as long as wide; disk relatively even with median longitudinal furrow very short and indistinct; punctures mostly round and slightly confluent, similar size to those of head; interstices smooth, mostly narrower than diameter of punctures except in median area, which may be triple as wide as diameter of punctures.

**Elytra** 0.93–1.00 times as long as wide; disk relatively even; punctures slightly and longitudinally confluent, of similar size to those of pronotum; interstices smooth, distinctly narrower than diameter of punctures.

**Legs** with tarsomeres IV strongly bilobed.

**Abdomen** cylindrical; paratergites very narrow and almost impunctate, present only in segment III, tergites and sternites totally fused in segments IV–VI, posterior margin of tergite VII with indistinct palisade fringe; punctures round, gradually becoming smaller and sparser posteriad; interstices smooth, mostly wider than diameter of punctures except those on basal impressions of tergites III–V, which may be distinctly narrower than half the diameter of punctures.

**Figures 14–20.**
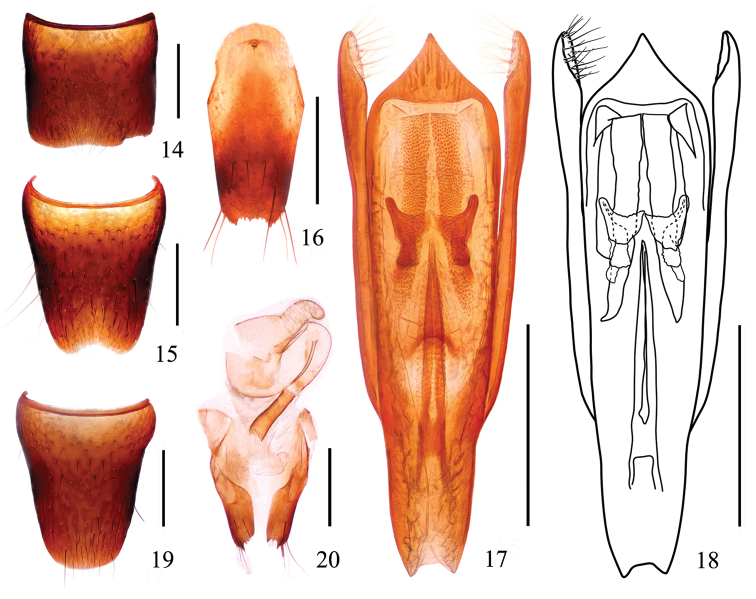
*Stenus yuyimingi*. **14** male sternite VII **15** male sternite VIII **16** male sternite IX **17, 18** aedeagus **19** female sternite VIII **20** valvifers and spermatheca. Scale bars: 0.25 mm.

***Male.*** Sternite VII (Fig. 14) with inconspicuous emargination at middle of posterior margin; sternite VIII (Fig. 15) with shallow emargination at middle of posterior margin; sternite IX (Fig. 16) with short apicolateral projections, posterior margin serrate. Aedeagus (Figs 17–18) apical sclerotized area triangular with a sharp projection at apex; median longitudinal bands each with ventral band very long, straight, narrowed apically; lateral longitudinal bands short; expulsion clasps each with anterior plate distinctly separated from the posterior plate, rounded apically; copulatory tube very long, rather slender, with basal chamber submembranous and main tube, the main tube gradually shrink, pointed apicaly; parameres as long as median lobe, each with about 19–22 setae on apico-internal margins.

***Female.*** Sternite VIII (Fig. 19) entire; spermatheca (Fig. 20) strongly sclerotized, swollen in the middle of spermathecal duct.

#### Distribution.

China (Zhejiang).

#### Remarks.

The new species can be easily distinguished from the Chinese members of the group by the coloration and sexual characters.

#### Etymology.

This species is named in honor of Mr. Yi-Ming Yu who collected some specimens of the new species.

### Stenus ovalis

Taxon classificationAnimaliaColeopteraStaphylinidae

Tang, Li & Zhao, 2005

Figs 5 , 6 , 21–24

Stenus ovalis Tang, Li & Zhao, 2005: 613.

#### Material examined.

**CHINA: Zhejiang**: 1♂, Longquan City, Fengyangshan N.R., Mihougu, 27°55’0.18’’N, 119°11’52.91’’E, near stream, 1116 m. 03.V.2016, Jiang, Liu & Zhou leg. (SHNU).

**Measurements.** BL: 4.2 mm, FL: 1.9 mm. HW: 0.77 mm, PL: 0.62 mm, PW: 0.56 mm, EL: 0.69 mm, EW: 0.67 mm, SL: 0.52 mm. Head 1. 14 times as wide as elytra, pronotum 1.10 times as long as wide, elytra 1.03 times as long as wide.

**Figures 21–24.**
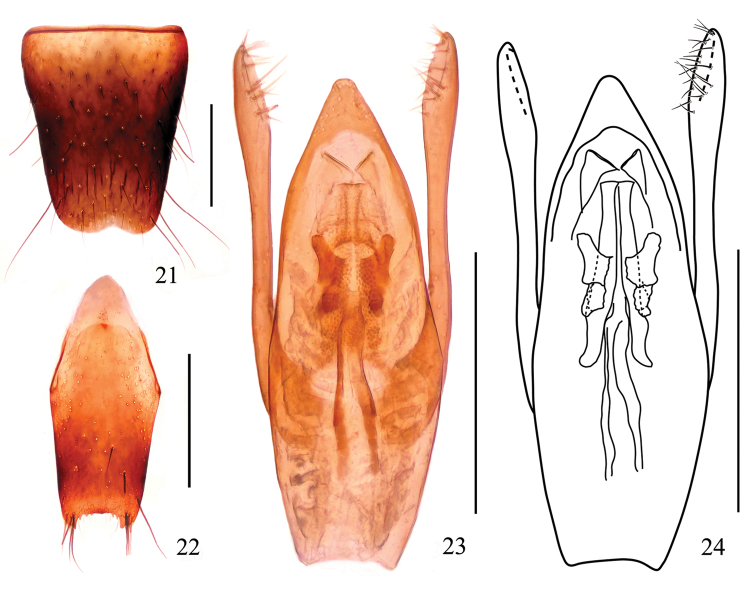
*Stenus ovalis*. **21** male sternite VIII **22** male sternite IX **23, 24** aedeagus. Scale bars: 0.25 mm.

***Male.*** Sternite VIII (Fig. 21) with a shallow emargination at middle of posterior margin; sternite IX (Fig. 22) with short apicolateral projections, posterior margin serrate. Aedeagus (Figs 23–24) apical sclerotized portion roundly projected at apex; median longitudinal bands each with ventral band long, narrowed apically; lateral longitudinal bands short; expulsion clasps each with anterior plate distinctly separated from the posterior plate, rounded apically; copulatory tube moderately long, rather stout, with basal chamber submembranous, main tube distinctly divided into basal tube and apical tube, the basal tube gently constricted near the middle, apical tube relatively thin; parameres longer median lobe, each with about 19 setae on apico-internal margins.

#### Distribution.

China (Zhejiang).

#### Remarks.

This is a new distributional locality of the species which is originally described from Wuyanling Nature Reserve of Zhejiang.

### Key to species of the *Stenus cirrus* group of Zhejiang

**Table:** 

1	Abdominal tergites III–VI with narrow to very narrow linear margination	***S. cirrus* L. Benick, 1940**
–	Only abdominal tergite III with complete paratergites, tergites, and sternites totally fused in segments IV–VI	**2**
2	Small species, BL = 2.3–3.1 mm, head and pronotum with distinct reticulation	**3**
–	Large species, BL = 3.5–5.0 mm, interstices of head and pronotum smooth	**4**
3	Interstices in median portions of head and pronotum much wider than diameter of punctures. Habitus: Figs 1, 2; sexual characters: Figs 7–13	***S. wuyanlingus* sp. n.**
–	Interstices in median portions of head and pronotum much narrower than diameter of punctures. Habitus: Fig. 4 (in Tang et al. 2008); sexual characters: Figs 20–24 (in Tang et al. 2008)	***S. shenshanjiai* Tang, Zhao & Puthz, 2008**
4	Elytra without orange spots. Habitus: Figs 3, 4; sexual characters: Figs 14–20	***S. yuyimingi* sp. n.**
–	Elytra with orange spots	**5**
5	Head distinctly wider than elytra	**6**
–	Head distinctly narrower than elytra. Aedeagus: Fig. 3 (in Puthz, 1998). BL = 3.5–4.7 mm	***S. guangxiensis* Rougemont, 1984**
6	Punctation of pronotum and elytra dense but not crowded. Habitus: Fig. 3 (in Tang et al. 2005); sexual characters: Figs 12–15 (in Tang et al. 2005). BL = 4.1–4.7 mm	***S. ovalis* Tang, Li & Zhao, 2005**
–	Punctation of pronotum and elytra very dense, crowded. Species best identified by their sexual characters	**7**
7	Punctation of elytra very densely crowded. Male: apical emargination 8th abdominal sternite very broad and shallow. Habitus: Fig. 1 (in Tang et al. 2008); sexual characters: Figs 7–11 (in Tang et al. 2008). BL = 3.7–5.0 mm	***S. zhulilongi* Tang & Puthz, 2008**
–	Punctation of elytra less dense, less crowded. Male: apical emargination of 8^th^ abdominal sternite narrower, rounded	**8**
8	Elytral spot longer, extending towards shoulder. Habitus: Fig. 2 (in Tang et al. 2008); sexual characters: Figs 12–15 (in Tang et al. 2008). BL = 3.8–5.0 mm	***S. lijinweni* Tang & Puthz, 2008**
–	Elytral spot shorter than half elytron length. Habitus: Fig. 3 (in Tang et al. 2008); sexual characters: Figs 16–19 (in Tang et al. 2008). BL = 3.7–5.0 mm	***S. jiulongshanus* Tang & Puthz, 2008**

## Supplementary Material

XML Treatment for Stenus wuyanlingus

XML Treatment for Stenus yuyimingi

XML Treatment for Stenus ovalis
